# Wear performance of different bulk-fill class II resin composite restorations: 3-year clinical evaluation

**DOI:** 10.1038/s41598-026-41420-7

**Published:** 2026-03-27

**Authors:** Badria Goda, Radwa I. Eltoukhy, Ashraf I. Ali, Salah Hasab Mahmoud

**Affiliations:** 1 Restorative Dentistry Department, Faculty of Dentistry, Horus University, New Damietta, Egypt; 2 Conservative Dentistry Department, Faculty of Dentistry, Mansoura University, El- Gomhoria Street, Mansoura, Egypt

**Keywords:** Health care, Materials science, Medical research

## Abstract

This study aimed to evaluate three-year qualitative and quantitative wear performance of a preheated body, an injectable and sonic bulk-fill resin composite, and compare it to that of a body bulk-fill composite placed in Class II cavities in a randomized clinical trial. For the clinical trial, 50 patients with a total of 80 Class II restorations in molars under normal occlusion were enrolled in this study. Restorations were placed by a single operator according to their manufacturers’ instructions, with (*n* = 20) for each material as follows: body bulk-fill (Filtek™ One Bulk-fill/Scotchbond Universal Adhesive), preheated body bulk-fill (Filtek™ One Bulk-fill/Scotchbond Universal Adhesive), injectable bulk-fill (G-ænial™ BULK Injectable/G-Premio BOND), and sonic bulk-fill resin composite (SonicFill3/OptiBond™ Universal). Immediately after placement, the restorations were finished/polished. Wear evaluation was performed qualitatively using modified Fédération Dentaire International (FDI) Criteria and quantitatively using Geomagic Control X software at baseline and at yearly intervals. The changes in FDI parameters during the three-year periods were analyzed and compared with the Chi-square test. For quantitative data, One Way ANOVA test was used to compare different studied groups over follow-up periods. The t-test was used to compare enamel surface volume loss with restoration volume loss within each studied group after one, two, and three years of follow-up. After 3 years, 78 restorations were recalled (97.5% recall rate) with 100% survival. All materials remained clinically acceptable regarding qualitative wear performance. For quantitative wear performance, ANOVA shows a significant difference between volume loss of the tested groups after one and two years, but not significantly different after three years. Mean volumetric wear (mm^3^) after 3 years was: body 0.020 ± 0.008, preheated body 0.018 ± 0.007, sonic 0.019 ± 0.009, and injectable 0.032 ± 0.010. T-test reveals insignificant difference between restoration and enamel volume loss within body, preheated body, and sonic-fill bulk-fill groups after three years. While injectable bulk fill volume loss significantly differs from enamel after one, two, and three years. All tested bulk-fill composites exhibited acceptable clinical performance qualitatively and quantitatively over 3 years. However, the injectable bulk-fill composite demonstrated greater volumetric loss than other materials, indicating lower long-term wear resistance under occlusal stress. All tested bulk-fill resin composites demonstrated excellent clinical performance and comparable wear resistance after three years. These findings support their reliable use in posterior Class II restorations, simplifying application without compromising longevity.

*Trial registration*: ClinicalTrials.gov Identifier NCT05861908, registered on 17 May 2023.

## Introduction

Continuous development of resin composite biomechanical properties increases its routine, well-established choice for posterior lesions in daily dental practice. Bulk-fill resin composite has been introduced as the preferred resin composite for posterior cavity restorations in scientific literature^1–3^. The introduction of bulk-fill composites has further simplified posterior restorations. Beyond reducing technique sensitivity, these materials allow placement in thicker increments (up to 4–5 mm), significantly reducing application time and light-curing cycles while maintaining adequate depth of cure and mechanical strength. Bulk-fill composites are particularly indicated for Class I and Class II restorations, where efficient placement and adequate adaptation are critical under clinical time constraints^4,5^.

Bulk-fill composites are available on the market in various viscosities, including high-viscosity bulk-fill (body bulk-fill), low-viscosity (flowable bulk-fill), and a special type of high-viscosity bulk-fill called sonic bulk-fill^6–8^. Sonic-fill allows bulk placement through a specialized handpiece that administers sonic energy at varying intensities. This energy application causes a significant drop in viscosity, which improves its adaptation^9,10^.

Since the introduction of bulk-fill resin composite in 2010, numerous studies have compared their properties with conventional counterparts, yielding conflicting results. Nonetheless, there is a consensus in scientific literature that bulk-fill composite effectively addresses the technique’s sensitivity concerns^11–14^. The high filler content typically found in commonly used body bulk-fill enhances their physico-mechanical properties and packing characteristics^15^. However, it also presented challenges in marginal and internal adaptation of the material to cavity walls, which could result in the formation of interfacial micro gaps and increased microleakage. To improve its marginal adaptation and clinical performance, new versions and methods were explored^16–19^.

Preheating is a commonly used approach to improve the adaptability of resin composites. Preheating reduces the composite’s viscosity by increasing the thermal motion of resin monomers and promoting their molecular separation. This process decreases the film thickness of the resin, enabling more efficient and uniform adaptation to cavity walls during placement. As a result, the reduction in marginal gaps observed following the preheating of composite resins is considered a justifiable and evidence-based improvement in restorative outcomes^20–22^.

In the past, low-viscosity bulk-fill was known to exhibit inferior mechanical properties compared to high-viscosity RCs, primarily due to the higher amount of filler particles in the latter. Consequently, their use was typically limited to small or minimally invasive cavity preparations, for repairing and sealing defective restorations, and as cavity base or liner in larger restorations. However, with the incorporation of nanofillers into this new generation of low-viscosity bulk-fill, their mechanical properties have been enhanced^23^. In addition, their self-leveling capacity guarantees an excellent adaptation to the cavity margins, while displaying a high DC and an improved stress-relieving capacity. Thus, they represent an interesting alternative for BF-RC restorations in the posterior region^6^.

Sonic bulk-fill resin composite is a specialized form of BBF-RC, wherein an air-driven handpiece is utilized to dispense the composite while applying sonic vibration. The manufacturer asserts that the material comprises a highly filled composite resin, along with modifiers that are activated by sonic energy, resulting in a reduction of the material’s viscosity by 84%. This reduction in viscosity facilitates better adaptation of the material during placement^24^. Following the deactivation of the sonic energy, the material viscosity increases, allowing for increased depths of cure^25^. Sonic bulk-fill has a depth of polymerization up to 5 mm and a polymerization shrinkage about1.6% with respect to volume^26^.

In addition to material formulation, the *adhesive strategy* plays a crucial role in the clinical performance of resin composites. Modern adhesive systems include *etch-and-rinse*, *self-etch*, and *selective enamel etching* protocols. The latter approach—used in this study—combines the advantages of both systems by applying phosphoric acid selectively to enamel margins, improving micromechanical retention while minimizing dentin overetching and postoperative sensitivity^27^.

Resin composite restorations in posterior teeth are subjected to a wide range of mechanical forces and chemical effects. If the forces applied to the resin composite restorations exceed the mechanical strength of the material, wear may occur^28,29^. The term ‘wear’ refers to net volume loss resulting from the interaction between at least two materials. However, intraoral wear is a highly complex phenomenon. The wear of both teeth and composite materials in the oral cavity is influenced by numerous factors, including food properties, temperature fluctuations, distribution of chewing forces, and pH levels^30^.

The wear behavior of resin composites is governed by several compositional and structural factors, including the type of resin matrix, filler particle size and loading, and the strength of the filler–matrix interface. Composites with smaller filler particles, higher filler content, and enhanced polymerization conversion typically demonstrate superior wear resistance, surface smoothness, and clinical longevity^31,32^. Despite continuous advancements in composite formulations and curing technologies, wear remains one of the leading causes of restoration failure and replacements within five to seven years of clinical service^33–35^. Therefore, evaluating the long-term wear performance of new bulk-fill resin composites under clinical conditions is critical for ensuring durable and functional posterior restorations^36^.

Most in vivo wear studies have generally used the subjective criteria of the US Public Health Service (USPHS) or FDI to qualitatively evaluate the clinical wear performance of bulk-fill resin composite restorations. A very few studies have investigated their wear quantitatively^37,38^. Human enamel has been considered ideal as a reference material in vivo study to compare and evaluate the wear tribology of restorative materials. The present study is in fact in continuation of these interests, but is intended to evaluate the qualitative and quantitative wear performance of a preheated body, an injectable and sonic bulk-fill resin composite restorative material, and compare it to that of a body bulk-fill composite in a 3-year randomized clinical trial study. The null hypotheses were that there is no significant difference in wear performance of different bulk-fill systems in Class II cavities during a three-year follow-up when evaluated qualitatively. Quantitatively, there is no significant difference in wear performance between different bulk-fill restorative systems and when compared to enamel wear after three years.

## **Methods**

### Study design

The present study was a prospective randomized double-blinded (Evaluators and patients) clinical trial, anticipating the four-arm parallel design in accordance with CONSORT 2010 guidelines for reporting trials. This study was ethically approved by the Faculty of Dentistry, Mansoura University, Dental Research Ethics Committee M01060421(6/4/2021). Moreover, it was registered in the clinical trial registration database (www.clinicaltrials.gov) under the identification number NCT05861908 (17/05/2023). The study was registered retrospectively because participant recruitment began before the initiation of the formal registration process. At that stage, the study was conducted as part of an academic research project within the faculty, and registration was completed later to ensure full transparency and adherence to ethical standards. It started on 9 May 2021 and was completed on 10 May 2024. All procedures involving human participants were in accordance with the ethical standards of the institutional and/or national research committee and with the Helsinki declaration. The research procedure was thoroughly explained to the participants. They signed a written informed consent form for the clinical study and its publication.

### Sample size calculation

Sample size calculation was based on the annual failure rate (AFR) of bulk-fill resin composites related to postoperative sensitivity and recurrence of caries, as reported by Durão et al. (2021). While it was conducted before research, without access to a paper with the same design of group allocation, we depended on the failure rate in one group and then had the same for the other studied groups. The calculation was performed using Epi Info™ version 7.2.4.0. based on a 1.2% prevalence, 95% CI, with an acceptable margin of error = 5. Accordingly, the total sample size needs to be 18 per group, but 20 were enrolled in each group to compensate for any unexpected dropouts.

### Participants’ recruitment and eligibility criteria

**Fig. 1:**
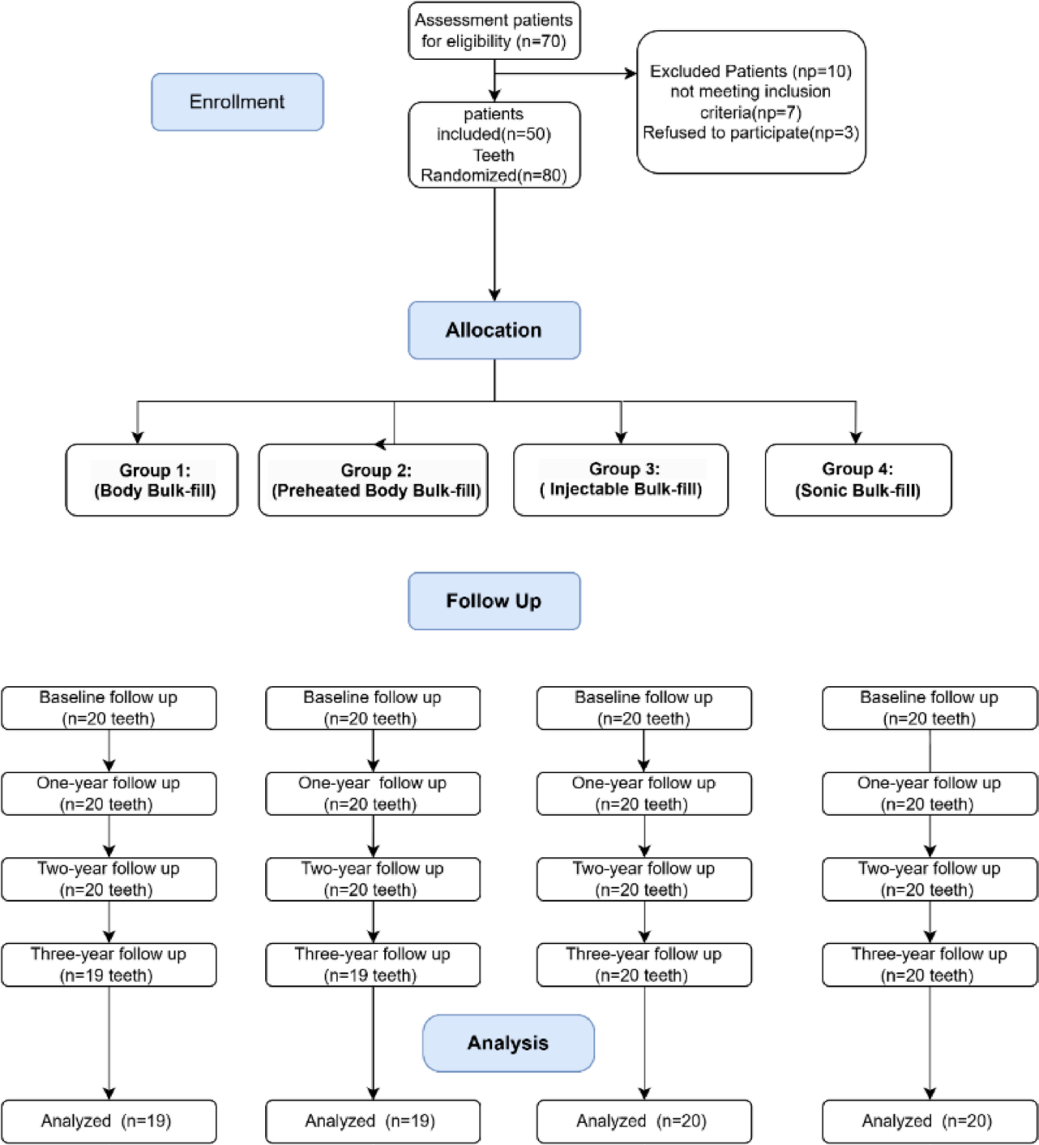
CONSORT flow chart.

Participants were recruited through social network advertisements and by hanging posters in the outpatient clinic at the Faculty of Dentistry, Mansoura University. In this study, the authors assessed 70 patients individually for eligibility. Inclusion and exclusion criteria are outlined in Table 1. Twenty patients were excluded due to either failing to meet the inclusion criteria or declining to come for follow-up visits. Finally, A total of 80 Class II carious lesions were restored in 50 patients. Each patient contributed one or two restorations, depending on the number of eligible teeth that met the inclusion criteria. When two restorations were placed in the same patient, they were assigned to different material groups according to the randomization schedule to reduce intra-patient bias. Fig. 1.

**Table 1:** Eligibility criteria.

Inclusion criteria	Exclusion criteria
1. Good general health 2. Patient aging 20–40 years 3. Patients available for follow-up visits 4. Good oral hygiene (patients with low and moderate caries risk according to the CAMBRA system. 5. Maxillary or mandibular first or second molars 6. Compound Class II (mesio-occlusal or disto-occlusal) 7. ICDAS 4 or 5 that diagnosed clinically and radiographically. 8. Normal response to a vitality test	1. Patients with orthodontic treatment 2. Severe or chronic periodontitis 3. Heavy bruxism 4. Sensitivity to resin-based material. 5. Patients on pregnancy or lactation 6. Chronic use of anti‑inflammatory drugs, analgesics, and/or psychotropic drugs 7. Teeth would need direct pulp capping 8. Teeth act as an abutment for a fixed or removable prosthesis 9. Occlusion of fewer than 20 teeth

To ensure consistency among participants, the severity of carious lesions was standardized according to ICDAS criteria (scores 4–5), and a bitewing radiograph was used to confirm limitation of caries to dentin without pulpal involvement. Patients with extensive caries, recurrent decay, or poor oral hygiene were excluded. All enrolled participants had moderate caries risk, as assessed by the CAMBRA system. Before restorative treatment, professional cleaning and individualized oral hygiene instructions were provided to establish a uniform baseline oral condition. In addition, patients’ oral hygiene and caries risk were reassessed during each follow-up period, and reinforcement of preventive measures was provided when necessary to maintain consistent oral health conditions throughout the study.

### Randomization, allocation, and blinding

A single operator using four different bulk-fill resin composite systems randomly restored 80 carious molars in 50 patients (23 females, 27 males). Randomization was done by generating 80 random numbers from 1 to 4 that represented the four restorative systems using a specific website (https://www.randomizer.org).

Group 1: Filtek™ One Bulk-fill/Scotchbond Universal Adhesive.

Group 2: preheated Filtek™ One Bulk-fill/Scotchbond Universal Adhesive.

Group 3: G-ænial™ BULK Injectable/G-Premio BOND.

Group 4: SonicFill3/OptiBond™ Universal.

The full description of all materials used is summarized, and the manufacturers’ instructions are presented in Table 2.

**Table 2:** Materials used in the study, their descriptions, and manufacturers’ instructions:

Material	Specification	Composition	Manufacture	(Lot number)	
Filtek™ bulk-fill	Nanohybrid Bulk-fill resin composite	Matrix: Aromatic urethane dimethacrylate (AUDMA), Urethane dimethacrylate (UDMA), Didodecyldimethylammonium bromide. Fillers: Silica, zirconia, ytterbium fluoride (76.5 wt% %, 58.4 vol% %	3 M-ESPE, St. Paul, MN, USA	(NC54613)	Placed directly into the cavity with a 4 mm thickness. *Heated Filtek™ Bulk fill* 1. The Therma-flo TM RC warming kit (Vista, Wisconsin, US) was turned on until it reached 68 C°. 2. Resin composite compule was placed in the heating chamber for 5 min. To reach the temperature of the warming device. 3. The compule was removed from the device, installed in the composite gun, and applied immediately into the cavity. The mean time between removing the compule from the device and light polymerization was approximately 15 s.
Single bond universal	Universal adhesive	10-Methacryloyloxydecyl dihydrogen phosphate (MDP), Bis-GMA, HEMA, silane, ethanol, water, initiator; pH 2.7.	3 M-ESPE, St. Paul, MN, USA	(01130 A)	1. Adhesive was applied to the prepared tooth and rubbed in for 20 s. 2. Adhesive was gently air-dried for approximately 5 s. To evaporate the solvent. 3. Adhesive was light-cured for 10 s.
G-ænial™ BULK Injectable	Injectable Nano hybrid Bulk-fill resin composite	Ethoxylated bisphenol-A dimethacrylate (Bis-EMA) 10–25%, Urethane dimethacrylate (UDMA) 0.2–0.5%, other dimethacrylates and additives (trade secret).absorber 0.2–0.5(Trade secret) Urethane dimethacrylate 0.2–0.5% dimethacrylate component0.2-0.5% (Trade secret)	(GC, United Kingdom)	(2009072)	The applicator tip was dispensed at the deepest cavity portion and was submerged for even resin distribution, avoiding bubble occurrence within the increment.
G-Premio BOND	Universal adhesive	10-Methacryloyloxydecyl dihydrogen phosphate (MDP), Methacryloyloxydecyl dihydrogen thiophosphate, 4-Methacryloyloxyethyl trimellitic acid, butylated hydroxytoluene, acetone, water, initiators, silica filler; pH 1.5.	GC Europe N.V., Leuven, Belgium/GC UK Ltd., Newport, UK	(2010081)	1. Adhesive was immediately applied to the prepared enamel and dentin surfaces using the disposable applicator. 2. It was left undisturbed for 10 s. after the end of application. 3. It dried thoroughly for 5 s. With oil-free air under maximum air pressure. 4. It was light-cured for 10 s.
SonicFill3	Sonic-activated Nanohybrid Bulk-fill resin composite.	Matrix: Ethoxylated Bis-GMA, Bis-GMA, TEGDMA. Fillers: Barium-alumino-silicate glass, silica, ytterbium fluoride (≈ 81.5 wt% %, 65.9 vol% %).	GC Europe N.V., Leuven, Belgium/GC UK Ltd., Newport, UK	(7520286)	1. The air pressure of the dental unit was adjusted to 50 PSI. 2. The dispensing rate/speed was adjusted to setting 3. 3. Its unidose tip was placed around one-half millimeter above the gingival margin to avoid air entrapment. 4. The foot pedal was activated to dispense material in the cavity. 5. The handpiece was slowly withdrawn as the cavity was filled, with the tip staying within the material to ensure good adaptation and avoid entrapment of air.
OptiBond™ Universal	Universal adhesive	HEMA, glycerol dimethacrylate, glycerol phosphate dimethacrylate, acetone, water, ethanol; pH 2.1.,	(Kerr Corp, Orange, CA, USA)	(7875665)	1. A generous amount of the adhesive was applied to the enamel/dentin surface and scrubbed with a brushing motion for 20 s. 2. It was dried with gentle air first and then medium air for at least 5 s. With oil-free air. The surface had having glossy, uniform appearance 3. It was light-cured for 10 s.

Numbers were written in well-sealed, opaque envelopes. Before the restorative procedures for each participant, volunteers who were not involved in the clinical study at any other time point selected envelopes. If the patient had more than one tooth, the tooth with the higher number was assigned to the first treatment according to the two-digit numbering system (teeth 11–48).

Two evaluators were responsible for restorative groups’ assessment after one, two, and three years using modified Fédération Dentaire International (FDI) Criteria. Since the operator could not be blind as the materials that had been used had different viscosities and delivery techniques, the study was double-blind (patient and Evaluators). *Patient blinding was maintained*, as all restorative procedures were performed under rubber dam isolation, and the materials had comparable shades and surface finishes after polishing. Patients were not informed about the type of composite used in their restorations.

### Clinical procedures

#### Isolation and cavity preparation

All patients’ teeth were cleaned with fluoride-free prophylaxis paste, and their shade was assessed before operative procedures. All operative procedures were performed under local anesthesia (Articaine HCL 4%) to prevent patients’ discomfort. After rubber dam isolation, conservative Class II cavities were performed using carbide pear-shaped burs (# 245, SS White Meta Dental Com, Korea) and spherical carbide burs (001/014) (MANI, INK. UTSUNOMIYA, TOCHIGI, JAPAN). Cavities were prepared under a constant copious water-cooling system using a high-speed contra-angled handpiece (Dentsply Sirona, York, PA, USA).

The cavity outline was restricted to the removal of carious tissue without beveling of the enamel margin. Remaining soft carious lesion, if present, was removed using a sharp excavator (Maillefer, Dentsply; Switzerland) or tungsten long round carbide burs (Mani, Ink. Utsunomiya, Tochigi, Japan) at a low-speed contra-angle handpiece (Dentsply Sirona, York, PA, USA). Cavity extended bucco-lingually according to caries extension without involving any cusps. Gingival margins, including sound enamel, were finished without violating biological depth.

After cavity preparation, the cavity was set to at least 4 mm depth, estimated using a periodontal probe. *A bitewing radiograph was taken* to confirm that the cavity depth and proximal margin did not violate the biological width. Teeth showing margins extending too close to the gingival crest, with pulpal exposure or having a cavity depth of less than 4 mm, were excluded. It was restored and withdrawn from the study and replaced by another eligible tooth from a different patient. Randomization was then repeated for the replacement case to maintain allocation balance. This ensured that all included cavities met the predefined depth and clinical inclusion criteria while keeping the total number of restorations constant.

Evaluation of the prepared floor depth was done by visual inspection using magnifying loupes 6X (Doctor Scope, Minam Optics Co, Korea) and confirmed by probing with a sharp explorer to assess the hardness underlying dentin. Discolored, but hard dentin was left in place on the cavity floor. All cavities’ internal line angles were finished using CD52-F fine diamond stones (MANI, INK. UTSUNOMIYA, TOCHIGI, JAPAN).

#### Placement of restoration

All cavities’ enamel margins were etched using 37%phosphoric acid for 15–20 s (N Etch, Ivoclar Vivadent). After etching, the tooth surface was rinsed thoroughly with an air–water syringe for 40 s to remove any residual phosphoric acid. The surface was then dried with oil-free compressed air for approximately 5 s, maintaining a moist dentin surface. Each universal adhesive was applied according to its assigned restorative group. The operator strictly followed the manufacturer’s instructions (Table 2).

All cavities were restored using pre-contoured sectional metallic bands that were fixed with a Garrison ring and adapted with plastic wedges (Composi-Tight 3D FusionTM, Garrison Dental Solutions, LLC, USA). Each bulk-fill resin composite was applied according to the manufacturer’s instructions (Table 2).

Sculpting and carving of composite resin was performed using a titanium-coated hand instrument (curved paddle “LRT”, Nordent, Bonneie lane, USA) and light-cured for 40 s. After the removal of wedges, ring, and the matrix, the restorations were cured for an additional 20 s. From the occlusal buccal and palatal/lingual direction.

Occlusion was checked using an articulating paper (Bausch, Nashua, NH, USA), then composite restorations were adjusted and finished using fine-grit diamond instruments (Diatech, Coltene, Switzerland). Finally, restorations’ embrasures were polished using discs (Sof‑Lex, 3 M‑ESPE, MN, USA) in the recommended order (coarse, medium, fine, and superfine) while occlusal surfaces were polished using rubber polishing instruments (One Gloss, Shofu, Kyoto, Japan), which are silicone-based rubber polishers containing alumina abrasive particles designed for one-step finishing and polishing of resin composites.

Polishing was carried out under continuous water cooling at low speed (10,000–12,000 rpm) with light, intermittent pressure, following the manufacturer’s instructions. The same protocol was applied to all groups to ensure consistency.

### Clinical evaluation

Two independent evaluators who did not participate in the study performed the evaluation (evaluator 1, evaluator 2). Before beginning the clinical assessment, an intra-evaluator and inter-evaluator agreement of at least 84% was requested. Calibration was conducted by clinically assessing 25 direct resin composite restorations from other volunteers not involved in the study. The level of agreement was verified using *Cohen’s kappa statistic (κ)*, which confirmed excellent reliability (κ = 0.86). For the *digital wear analysis*, STL datasets from 10 randomly selected restorations were reanalyzed by the same and a second examiner to assess intra- and inter-rater reliability using the *intraclass correlation coefficient (ICC*,* two-way mixed model*,* absolute agreement)*. Both ICC values were above 0.90, confirming excellent consistency of the volumetric measurement protocol.

A standardized case report form was used for each restoration to record the modified FDI parameters at all evaluation intervals. Each restoration was assigned a *coded identification number* that did not reveal its restorative material. After each observation, the completed coded forms were collected and reviewed by a research coordinator who was not involved in the evaluations. This procedure ensured that both evaluators remained *blinded to group allocation* throughout all follow-up periods, maintaining the integrity of the double-blind design. For qualitative wear analysis, direct evaluation was performed using modified FDI criteria^39^, As illustrated in Table 3.

**Table 3:** Selected modified FDI criteria for evaluation.

	Occlusal contour and wear qualitatively
Clinically excellent/very good	a.1 Physiological wear equivalent of enamel.
Clinically good (after polishing, probably very good)	a.2 Normal wear is only slightly different from that of enamel.
Clinically sufficient/satisfactory (minor shortcomings, no unacceptable effects, but not adjustable w/o damage to the tooth).	a.3 Different wear rate than enamel but within the biological variation.
Clinically unsatisfactory (repair for prophylactic reasons)	a.4 Wear considerably exceeds normal enamel wear; or occlusal contact points are lost.
Clinically poor (replacement necessary)	a.5 Wear is excessive.

Regarding wear analysis quantitatively, one pre-functional reference quadrant impression (directly after finishing and polishing), and four follow-up functional impressions (baseline (one week), one, two, and three years) were taken using additional silicon impression material (Zhermack Elite HD, Zhermack SpA, Badia Polesine (RO), Italy). Replicas were generated by pouring these impressions with stone containing resin particles (Elite Master, Zhermack SpA, Badia Polesine (RO), Italy) using a vibrator (Patterson 3-Speed Heavy Duty 4” Vibrator, Patterson Dental, Saint Paul, MN).

**Fig. 2:**
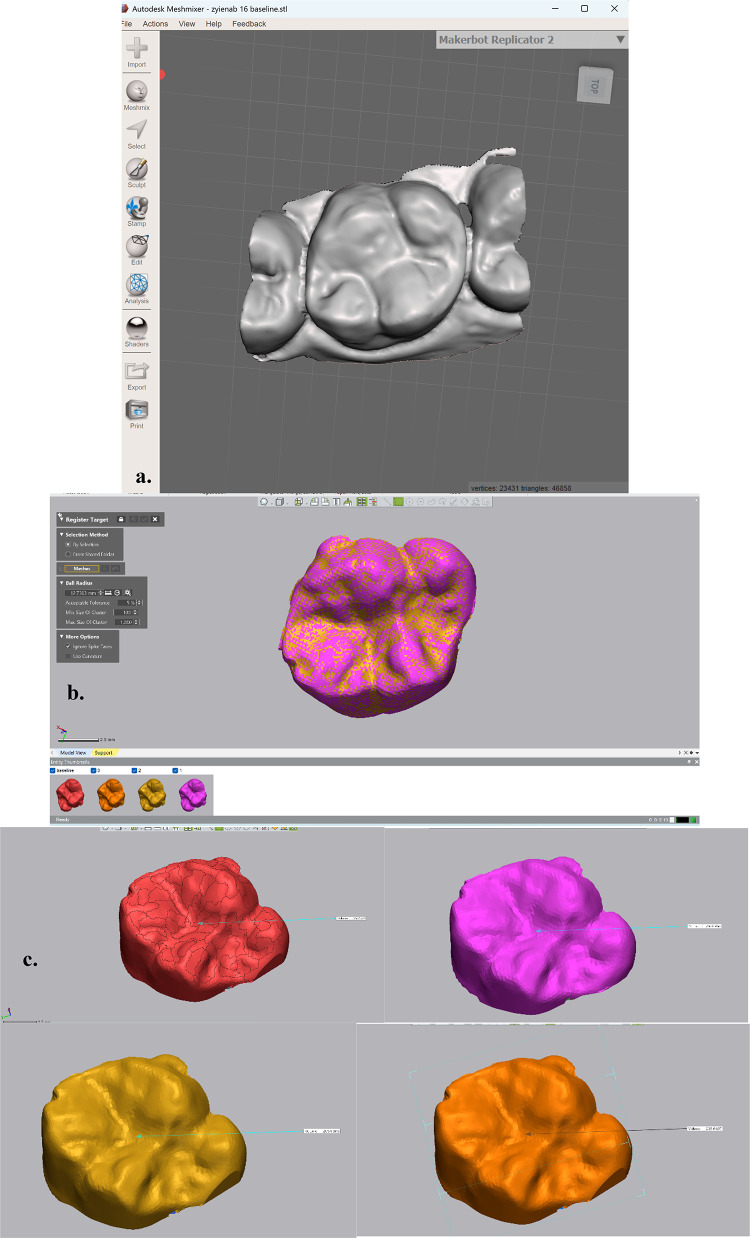

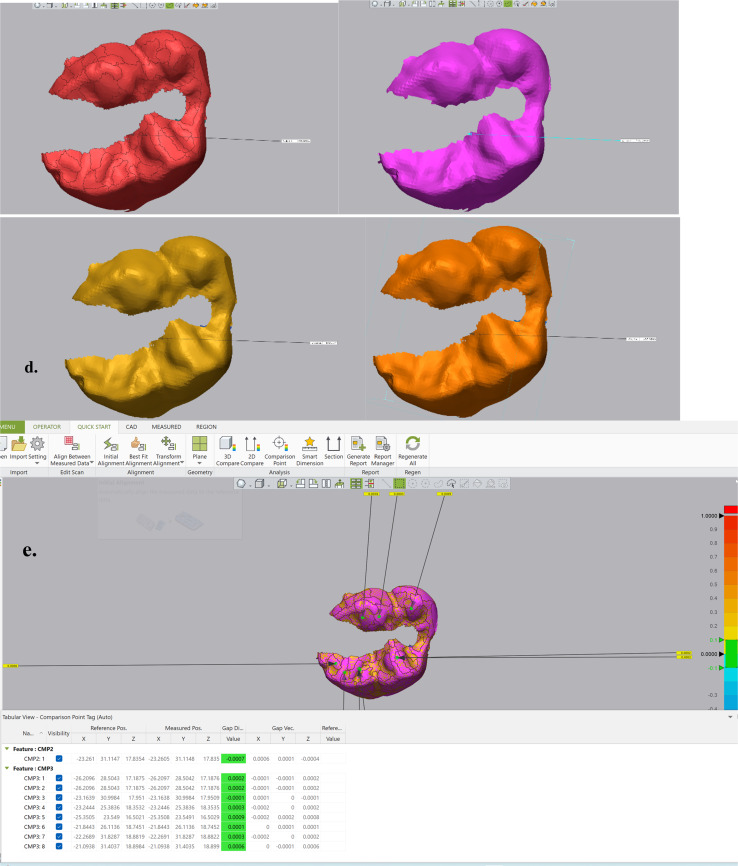
Steps of wear calculation. (**a**) STL file for scanned subjects. (**b**) superimposed and best-fit alignment of Color-coded 3D models of occlusal surfaces at different recall periods using Geomagic Control X Software. (**c**) Separated 3D volumes with corresponding TSV measurements. (**d**) Separated 3D volumes with corresponding ESV measurements. (**e**). Comparison point for overlapped subject here dimensionally.

Stone models were scanned three-dimensionally using a 3D laser scanner (InEos X5, Dentsply Sirona, York, PA, USA). The obtained Standard Tessellation Language (STL) files were analyzed using MeshMixer software (MeshMixer Autodesk, USA) that converted acquired meshes into closed solid ones after making plain cuts to the excluded unexamined objects. (Fig. 2) Standard Tessellation Language file of prefunctional and each follow-up interval was superimposed and aligned using Geomagic control X software (3D Systems, Darmstadt, Germany). The operator identified sections of the mesh that are least likely to have changed as buccal and lingual surfaces of reference, followed by fine registration using the iterative closest point (ICP) algorithm. This method minimizes distortion at the restoration interface and ensures accuracy of volumetric comparison between baseline and follow-up scans. “Reference best-fit alignment”. The statistical mode for images was used to quantify the total surface volume (TSV) baseline and each follow-up in mm^3^. Total surface volume loss (TSVloss) was calculated by subtracting the follow-up image from base baseline.

The edit function was utilized to select and remove the restoration area from superimposed images. Enamel surface volume (ESV) was obtained. The ESV loss was obtained by subtracting two images. Finally, restoration volume loss was calculated by subtracting ESV loss from TSV loss (Fig. 2). 

### Statistical analysis

Statistical analysis was conducted utilizing SPSS 20^®^ (Statistical Package for Social Science, IBM, USA) and Microsoft Excel 2016 (Microsoft Corporation, USA). Survival data were analyzed using a *z-test for proportions* to compare the survival rates of restorations at baseline, one-, two-, and three-year follow-ups. This test was used to determine whether the observed differences in survival proportions across the follow-up intervals were statistically significant. Qualitative data obtained were presented as frequency & percentages, where comparisons were performed by the Chi-square test. For quantitative data, the Way ANOVA test was used to compare different studied groups after one, two, and three years. The t-test was used to compare enamel surface volume loss with restoration volume loss within each studied group after one, two, and three years of follow-up.

## Results

Most of the participants in the study are males (54%). The mean age of the participants was 30 ± 10 years. Eighty restorations were made, 20 for each group. The distribution of the restorations is illustrated in Table 4.

**Table 4:** Characteristics of the participants and cavities.

Characteristics		Number of restorations			
		Body bulk-fill	Preheated body bulk-fill	Injectable bulk-fill	Sonic bulk-fill
Participants	Female	9	5	7	8
	Male	11	15	13	12
Tooth distribution	Upper 1st Molar	3	5	7	5
	Upper 2nd Molar	7	6	2	3
	Lower 1st Molar	8	4	9	10
	Lower 2nd Molar	2	6	2	2
Cavity depth	4 mm	13	14	11	13
	˂4 mm	7	6	9	7

Statistical comparison using the z-test for proportions revealed no significant difference (*p* > 0.05) in survival rates across all follow-up intervals, as all restorations demonstrated 100% survival up to two years and 100% survival at three years, with only one patient (two restorations) lost to follow-up due to relocation (*recall rate was 97.5%*).

**Fig. 3:**
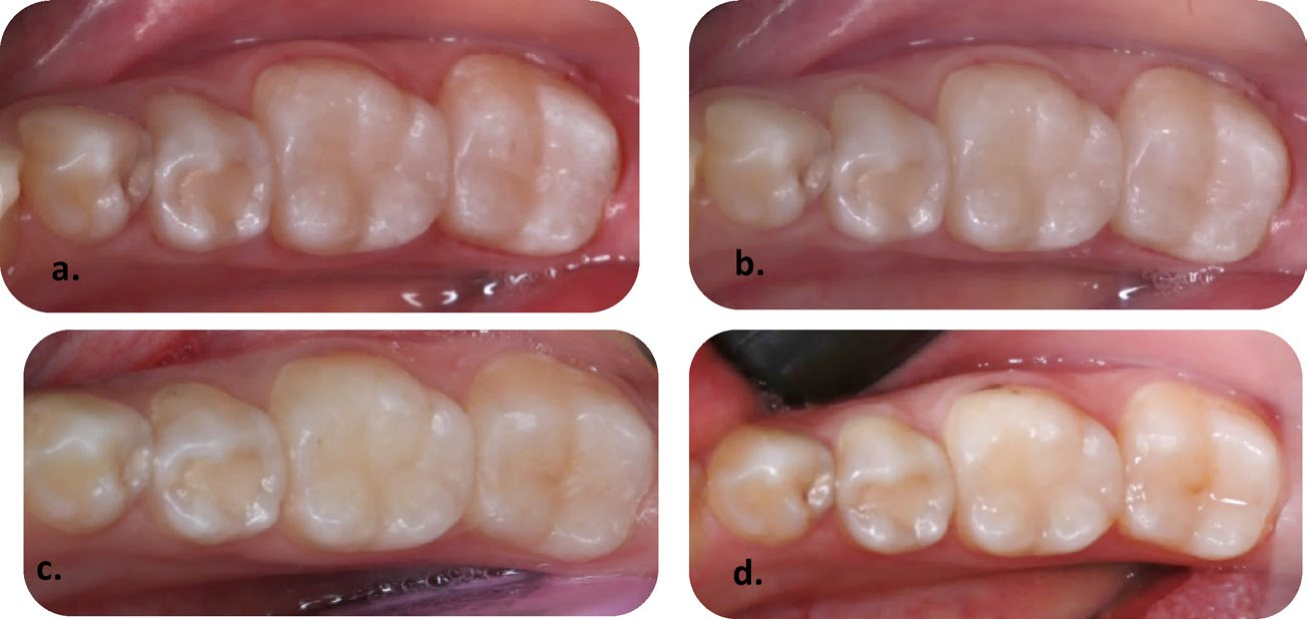
Representative clinical photographs showing the Distal Class II body bulk-fill restoration tooth no. 36. (**a**) baseline follow-up is showing clinically excellent restoration, (**b**) one-year follow-up is showing clinically excellent restoration, (**c**) two-year follow-up is showing clinically excellent restoration, (**d**) three-year follow-up is showing clinically excellent restoration.

**Fig. 4:**
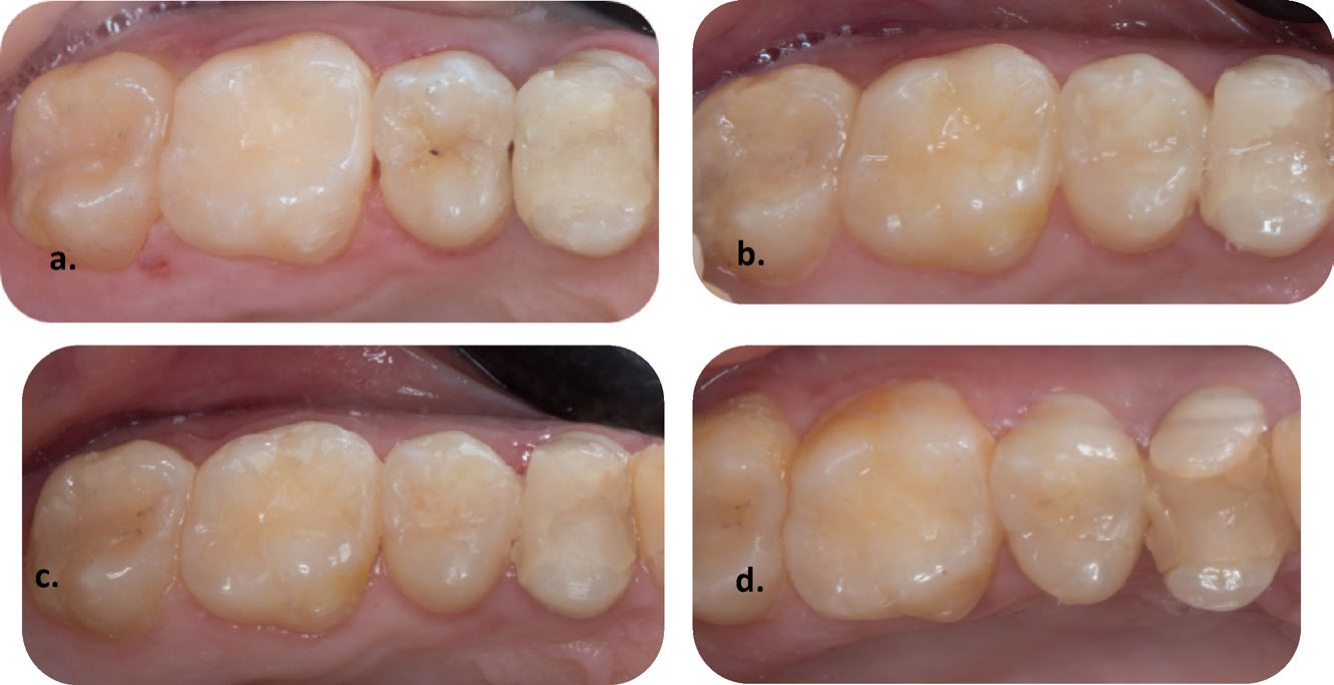
Representative clinical photographs showing the Mesial Class II Preheated body bulk-fill restoration of tooth no. 16. (**a**) baseline follow-up is showing clinically excellent restoration, (**b**) one-year follow-up is showing clinically excellent restoration, (**c**) two-year follow-up is showing clinically excellent restoration, (**d**) three-year follow-up is showing clinically good restoration.

**Fig. 5:**
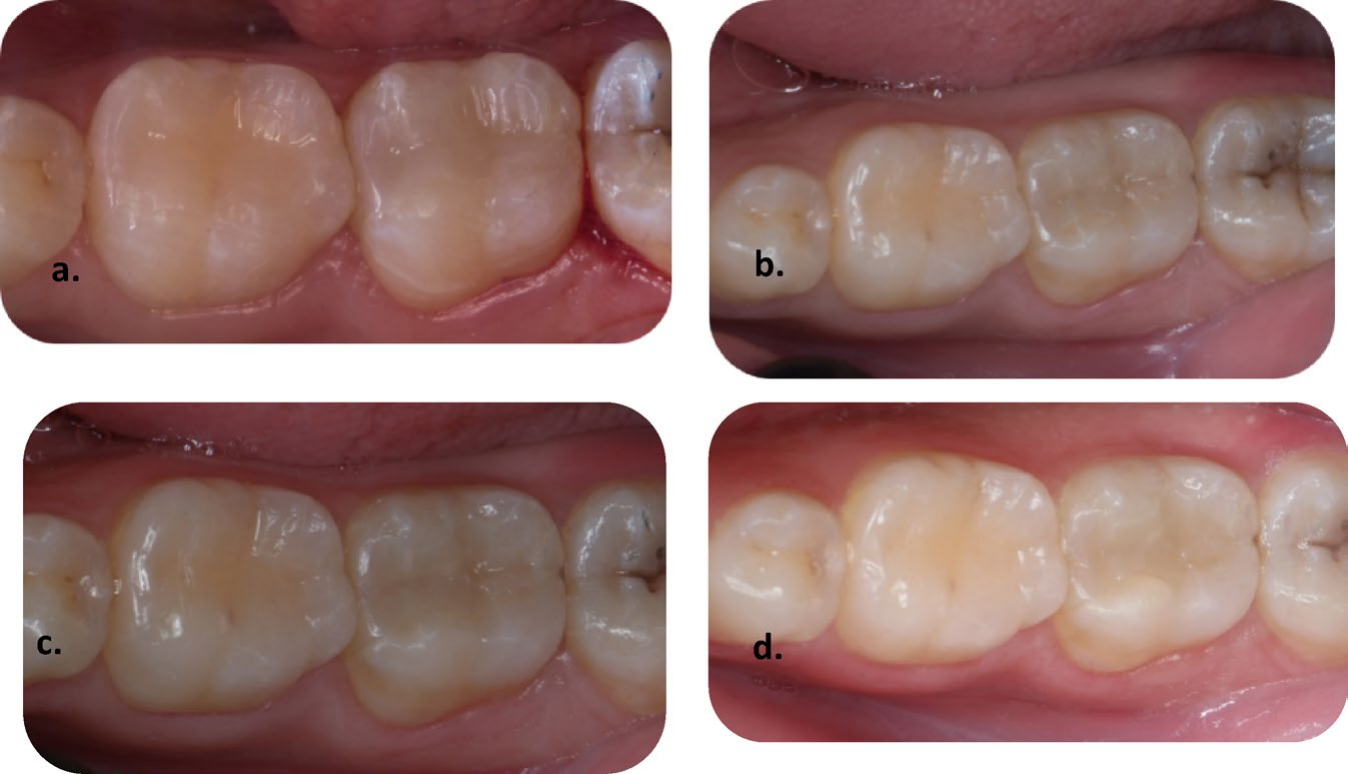
Representative clinical photographs showing a Mesial Class II tooth, an Injectable bulk-fill restoration of tooth no. 37. (**a**) baseline follow-up is showing clinically excellent restoration, (**b**) one-year follow-up is showing clinically excellent restoration, (**c**) two-year follow-up is showing clinically excellent restoration, (**d**) three-year follow-up is showing clinically good restoration.

**Fig. 6:**
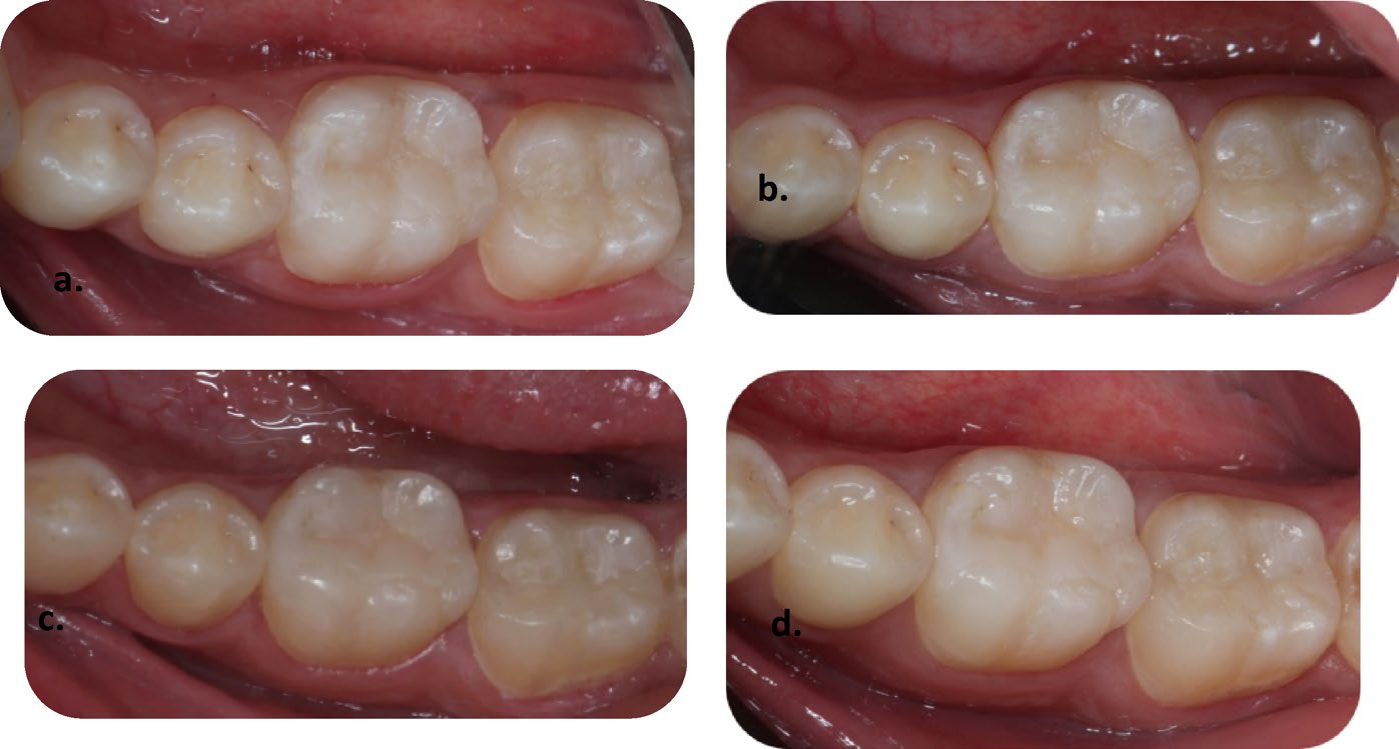
Representative clinical photographs showing Mesial Class II Sonic-fill bulk-fill tooth no. 46. (**a**) baseline follow-up is showing clinically excellent restoration, (**b**) one-year follow-up is showing clinically excellent restoration, (**c**) two-year follow-up is showing clinically excellent restoration, (**d**) three-year follow-up is showing clinically excellent restoration.

Representing digital clinical photographs after one, two, and three years of follow-up are illustrated in Figs. 3, 4, 5 and 6.

Figure 3 illustrates representative clinical photographs of the distal Class II body bulk-fill restoration on tooth no. 36 at baseline, one-year, two-year, and three-year follow-up, which shows clinically excellent results.

Figure 4 illustrates representative clinical photographs of the mesial Class II Preheated body bulk-fill restoration on tooth no. 16 at baseline, one-year, and two years, which shows clinically excellent results. Where three-year follow-ups show clinically good results.

Figure 5 illustrates representative clinical photographs of a mesial Class II tooth, an Injectable bulk-fill restoration on tooth no.37 at baseline, one-year, and two-year, which show clinically excellent results. Where three-year follow-ups show clinically good results.

Figure 6 illustrates representative clinical photographs of the mesial Class II Sonic-fill bulk-fill on tooth no.46 at baseline, one-year, two-year, and three-year follow-up, which show clinically excellent results.

Frequency and percentages of different scores of all groups are presented in Table 5. Chi-square test was used to compare the different intervals within each group (Intragroup comparison) and between the different groups at each time interval (Intergroup comparison).

**Table 5:** Frequency and percentage of recorded scores at baseline, one, two, and three years regarding the tested properties of all groups.

Occlusal contour and wear (Qualitatively)						
		Baseline	2 years	2 years	3 years	*p* value
Body bulk-fill	Score 1	20 (100)	20 (100)	20 (100)	17 (89.5)	0.91
	Score 2	0 (0)	0 (0)	0 (0)	2 (10.5)	
Preheated body bulk-fill	Score 1	20 (100)	20 (100)	20 (100)	17 (89.5)	0.91
	Score 2	0 (0)	0 (0)	0 (0)	2 (10.5)	
Injectable bulk-fill	Score 1	20 (100)	20 (100)	20 (100)	18 (90)	0.11
	Score 2	0 (0)	0 (0)	0 (0)	2(10)	
Sonic bulk-fill	Score 1	20 (100)	20 (100)	20 (100)	18 (90)	0.11
	Score 2	0 (0)	0 (0)	0 (0)	2(10)	
*p* value		–	–	–	1.00	

*p* < 0.05 is considered statistically significant. Clinically relevant differences are those in which wear variation exceeds the normal biological range of enamel wear or affects occlusal contour integrity.

Regarding occlusal contour and wear, the chi-square test revealed insignificant differences within intragroup and intergroup comparisons across all time intervals. Within the qualitative method, all groups revealed a 100% score of 1 at all-time intervals for intragroup comparison concerning body, preheated body, injectable, and sonic bulk-fill resin composite. Moreover, their intergroup comparison detected no significant difference between all groups at baseline, one, two, and three-year follow-up.

**chart 1:**
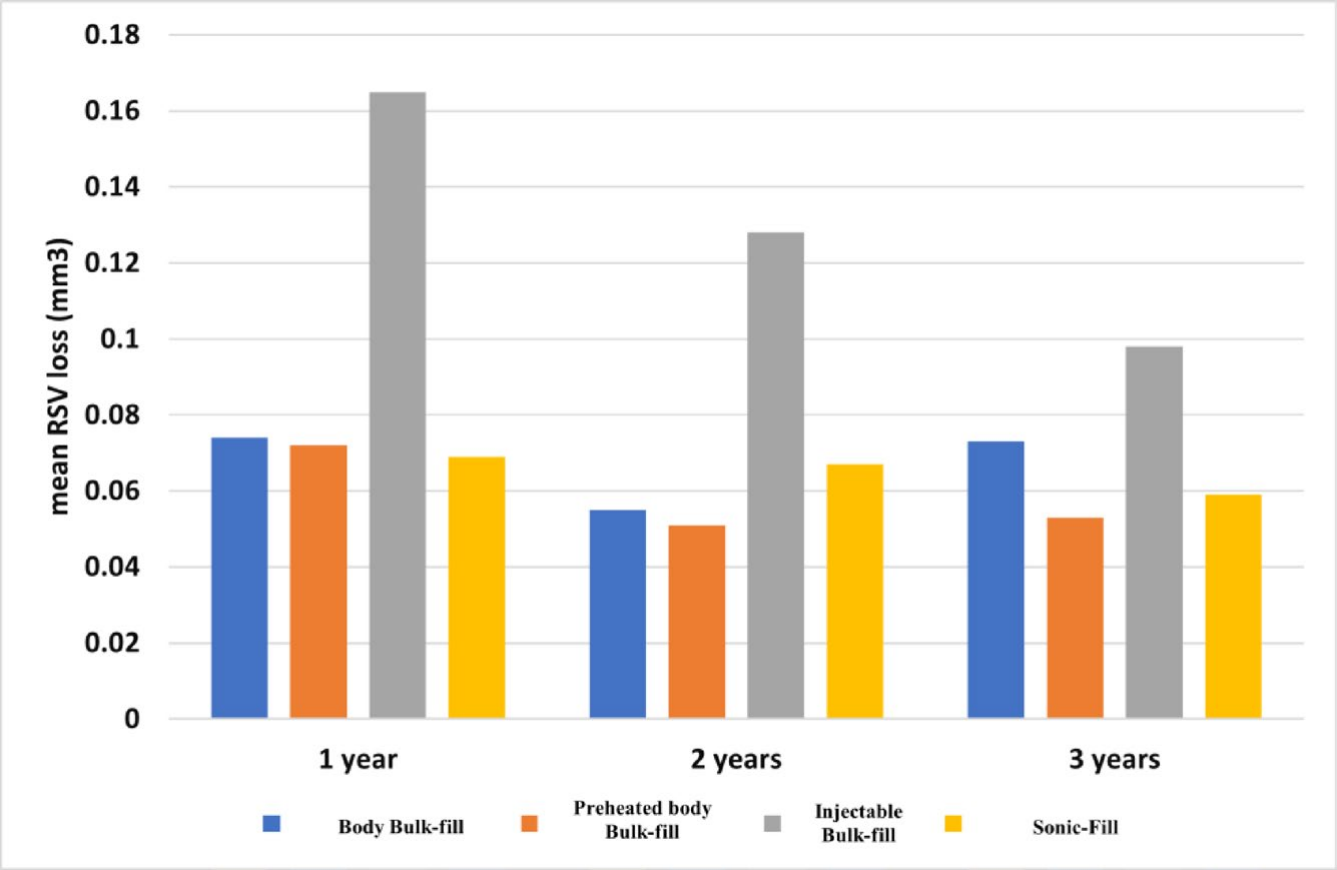
Clustered column chart showing mean RSV loss (mm^2^) for studied groups along follow-up periods.

For quantitative data, the Shapiro-Wilk Test showed that the data followed a normal distribution. Parameters described as mean (standard deviation), then one-way ANOVA test reveals insignificant difference between body bulk-fill and all tested groups after one and two years follow up except injectable bulk-fill. After three years, there is no significant difference in RSV loss of body bulk-fill and all tested groups (Table 6, chart 1. ).

**Table 6:** Comparison of RSV loss between studied groups and during follow-up.

		Body bulk-fill	Preheated body bulk-fill	Injectable bulkfill	Sonic bulk-fill	Test of significance	η^2^ (effect size)	Within-group significance
RSV loss	1 year	0.074 ± 0.015	0.072 ± 0.02	0.165 ± 0.09	0.069 ± 0.023	F = 8.64 *P* = 0.001*	0.25	P1 = 0.919 P2 = 0.001* P3 = 0.842 P4 = 0.001* P5 = 0.922 P6 = 0.001*
	2 years	0.055 ± 0.029	0.051 ± 0.014	0.128 ± 0.04	0.067 ± 0.017	F = 016 *P* = 0.001*	0.20	P1 = 0.754 P2 = 0.001* P3 = 0.354 P4 = 0.001* P5 = 0.218 P6 = 0.001*
	3 years	0.073 ± 0.07	0.053 ± 0.012	0.098 ± 0.04	0.059 ± 0.01	F = 2.11 *P* = 0.117	0.08	P1 = 0.311 P2 = 0.210 P3 = 0.473 P4 = 0.027* P5 = 0.765 P6 = 0.053
P= Pairwise comparison		0.667	0.01*	0.058	0.471			
		p_a_ = 0.083 p_b_ = 0.970 p_c_ = 0.560	p_a_ = 0.003* p_b_ = 0.032* p_c_ = 0.798	p_a_ = 0.254 p_b_ = 0.04* p_c_ = 0.118	p_a_ = 0.687 p_b_ = 0.319 p_c_ = 0.434			

Mean (± SD) volumetric loss (mm^3^) of composite restorations over the follow-up period. F, One Way ANOVA test; p1, difference between Body Bulk-fill versus Preheated Body Bulk-fill; p2, difference between Body Bulk-fill versus Injectable BULKFILL; p3, difference between Body Bulk-fill versus Sonic Bulk-fil; p4, difference between Preheated Body Bulk-fill versus Injectable BULKFILL; p5,difference between Preheated Body Bulk-fill versus Sonic Bulk-fill; p6, difference between Injectable BULKFILL versus Sonic Bulk-fill; Pa, difference between 1 year versus 2 years; pb, difference between 1 year versus 3 years; pc, difference between 2 versus 3 years. *p* < 0.05 is considered statistically significant. Clinically relevant differences are those in which wear variation exceeds the normal biological range of enamel wear or affects occlusal contour integrity.

η^2^ = partial eta squared (effect size for ANOVA); Interpretation: small = 0.2, medium = 0.5, large = 0.8.

ANOVA test reveals insignificant differences between one, two, and three years of RSV loss of body bulk-fill, injectable bulk-fill, and sonic-fill (*p* value: 0.667, 0.058, and 0.471, respectively). Volume loss of preheated body bulk-fill, after one, two, and three years, is significantly different (*p* value: 0.01). Clinically, this indicates that while early differences in wear among materials were detectable, these did not translate into long-term clinically relevant discrepancies after three years of service (Table 6, Chart 7).

Effect size analysis supported the significance of early wear differences. The ANOVA tests showed large (η^2^ = 0.25) and moderate (η^2^ = 0.20) effects after one and two years, but a small effect (η^2^ = 0.08) after three years, suggesting convergence in long-term performance.

Regarding comparison of ESV loss with RSV loss, the T-test compared all tested groups with ESV loss and revealed a significant difference after one year, which suggests comparable clinical wear behavior to natural enamel. After two years, injectable bulk-fill and sonic-fill RSV loss revealed a significant difference compared to ESV loss (*p* value: 0.001, 0.014, respectively) while only injectable bulk-fill RSV loss significantly differed from ESV loss after three years (*p* value: 0.001), indicating a clinically relevant reduction in wear resistance over time (Table 7).

**Table 7:** Comparison of ESV loss versus REV loss within each studied group after one, two, and three years of follow-up.

			Mean	Std. deviation	*p* value	Cohen’s d (effect size)
Body bulk-fill	1 Year	ESV loss	0.0372	0.02179	0.001*	1.96
		RSV loss	0.0743	0.01544		
	2 Years	ESV loss	0.0432	0.02067	0.366	0.47
		RSV loss	0.0554	0.02913		
	3 Years	ESV loss	0.0508	0.02949	0.468	0.35
		RSV loss	0.0733	0.07400		
Preheated body bulk-fill	1 Year	ESV loss	0.0392	0.01142	0.004*	1.96
		RSV loss	0.0720	0.02102		
	2 Years	ESV loss	0.0737	0.09665	0.497	0.31
		RSV loss	0.0514	0.01498		
	3 Years	ESV loss	0.0360	0.02158	0.057	0.93
		RSV loss	0.0532	0.01217		
Injectable bulkfill	1 Year	ESV loss	0.0392	0.00941	0.002*	1.73
		RSV loss	0.1649	0.09362		
	2 Years	ESV loss	0.0506	0.01493	0.001*	2.20
		RSV loss	0.1285	0.04272		
	3 Years	ESV loss	0.0399	0.00861	0.001*	1.75
		RSV loss	0.0983	0.04095		
Sonic bulk-fill	1 Year	ESV loss	0.0453	0.01300	0.02*	1.17
		RSV loss	0.0698	0.02354		
	2 Years	ESV loss	0.0424	0.01846	0.014*	1.42
		RSV loss	0.0673	0.01788		
	3 Years	ESV loss	0.0459	0.01279	0.096	0.80
		RSV loss	0.0591	0.01889		

Mean (± SD) volumetric loss (mm^3^) of composite restorations and enamel surfaces over the follow-up period. T-test was used, p*: significant difference, *p*-value = 0.05, d = Cohen’s d for paired comparisons. *Interpretation thresholds*: small = 0.01–0.06; medium = 0.06–0.14; large ≥ 0.14.

Pairwise comparisons (Cohen’s d) indicated that the injectable bulk-fill group consistently exhibited large effect sizes (d > 1.5) versus enamel, confirming the clinical relevance of its greater wear. In contrast, other groups showed small-to-moderate effect sizes (d < 0.8), reflecting differences unlikely to be of clinical concern.

## Discussion

The present randomized clinical trial evaluated the three-year qualitative and quantitative wear performance of four bulk-fill resin composites—body, preheated body, injectable, and sonic—in Class II restorations. After three years, all restorations remained clinically acceptable according to modified FDI criteria, with a 100% survival rate, however quantitative analysis, revealed differences in wear behavior among materials. The injectable bulk-fill composite exhibited significantly greater volumetric loss compared to enamel and the other bulk-fill materials, particularly during the first two years of service. So, the first hypothesis of the current study is accepted, while the second null hypothesis is rejected.

The magnitude of variation of other groups remained within the biological range of enamel wear and was therefore not clinically significant. This aligns with the FDI qualitative evaluation, where all restorations maintained clinically excellent ratings throughout the study. These observations highlight that statistical significance does not necessarily imply clinical importance, and the overall performance of body, preheated, and sonic bulk-fill composites remains clinically satisfactory after three years.

The present findings align with previous short-term clinical evaluations reporting satisfactory wear resistance for preheated and sonic bulk-fill composites, Durão et al.^40^ Similar results were also observed by Nascimento Poubel et al.^21^ Who found that thermally modified high-viscosity composites exhibit improved flow, adaptation, and polymerization efficiency, resulting in reduced wear. Conversely, the higher wear recorded for the injectable bulk-fill material agrees with laboratory studies indicating that flowable composites generally have lower filler loading and reduced mechanical strength, leading to greater material loss under occlusal stress^39^.

The differences in wear performance observed in this study can be attributed to variations in filler size, volume fraction, and matrix formulation among the tested materials. The emerging dental research innovations in filler technologies now include the maximized use of nanoscale filler particles, owing to their potential to directly affect most composite properties, including wear resistance^41^. Resin composite with a higher filler load and stronger filler–matrix bonding exhibit improved stress distribution and reduced microcracking under occlusal load, leading to lower material loss. The body, preheated and sonic bulk-fill composites in this study contained higher filler percentages and achieved a higher degree of conversion, which may explain their favorable wear resistance. Conversely, the injectable composite, characterized by lower filler loading and higher resin content, showed greater volumetric loss, consistent with laboratory evidence linking reduced filler density to increased wear and surface degradation^31,32^.

Quantitative wear patterns cannot be precisely studied without sophisticated indirect methods, and clinical qualification can only be obtained in long-term clinical studies. Therefore, prospective randomized clinical trials (RCTs) to evaluate the wear performance of bulk-fill resin composite with different placement techniques were performed.

Assessing wear can be challenging for examiners because it’s typically not uniform across all restoration surfaces, especially in cases where the entire occlusal surface has been reconstructed. In these instances, comparing restoration wear with the enamel wear and comparing baseline with follow-ups, as recommended by modified FDI, was found to be helpful^42^.

Body and preheated body bulk-fill resin composite achieved their depth of polymerization by incorporating a high-molecular-weight AUDMA monomer and integration of AFM with a dimethacrylate monomer. As a resin composite is a viscoelastic material, preheated body BF-RCs reduce the viscosity of dimethacrylate and increase radical mobility. This effect leads to additional polymerization and a higher degree of conversion^43–45^. This participation formed a network, reducing the number of reactive groups in the resin, thereby increasing the degree of conversion and wear resistance. Based on studies in the literature, it seems evident that not only the filler properties and the polymer matrix, but also the photopolymerization time have a role in improving the mechanical properties of the resin composite^46,47^. Proper polymerization increases the mechanical properties, fracture, and wear resistance^48,49^. Previous studies have shown that wear resistance is strongly influenced by the filler–matrix interaction and not solely by preheating or viscosity modifications^50,51^.

On the other hand, injectable bulk-fill resin composite features a round-shaped filler morphology, resulting in high flexural strength and hardness compared to irregularly shaped fillers. But it has lower filler loading and flowable consistency, which, while advantageous for adaptation and ease of placement, compromises its mechanical strength under occlusal stress. Furthermore, the resin matrix may be more prone to hydrolytic degradation and fatigue over time, leading to matrix-filler interface breakdown and accelerated surface loss^52,53^. This explains the significant difference between injectable bulk-fill and other tested groups in volume loss after three years.

The findings highlight the need for careful material selection in stress-bearing restorations, particularly where long-term wear resistance is critical. Although statistical differences in wear were found during early follow-ups, the accompanying effect sizes clarify that only the injectable bulk-fill composite exhibited a clinically meaningful magnitude of wear (large d values). The body, preheated, and sonic bulk-fill groups showed small-to-moderate effects, indicating minimal practical impact despite some statistical variation. These findings emphasize that effect size analysis complements *p*-values by distinguishing clinically important outcomes from statistically detectable but negligible ones.

Exclusion of patients with parafunctional habits, careful consideration of biting forces, and meticulous finishing and polishing of restorations were speculated to be the reasons for the proper occlusal wear compared to enamel wear. Additionally, ensuring adequate light polymerization for bulk-fill resin composite primarily enhanced the degree of polymerization and the physical properties^54^.

In any indirect quantitative method used to quantify clinical wear, it is crucial to consider the effects of impression and replica materials to ensure the accuracy of the scanning system. In this study, the 3D laser scan method utilized had an accuracy of 10 μm, and the reported error for the replication procedure was between 8.2 and 9 μm, totaling approximately 20 μm. Therefore, an upper limit of 15–20 μm was deemed acceptable for the superposition routine in this study. Since this error component was applied consistently across all restorations, the method can be considered an acceptable means of detecting differences between the groups^55^.

The present trial was *registered retrospectively* after the onset of patient recruitment. At that stage, the study was conducted as part of an academic research project within the faculty, and registration was completed later to ensure full transparency and adherence to ethical standards. To mitigate this limitation, all study procedures, outcome measures, and analyses strictly followed the protocol approved by the Research Ethics Committee of the Faculty of Dentistry, Mansoura University (M01060421). No deviations from the intended study design occurred, and all results were reported according to the CONSORT checklist.

Although the study followed a randomized controlled design, its parallel structure and relatively modest sample size represent notable limitations. This design naturally introduces interpatient variability, which may slightly restrict the generalizability of the findings^56^. Nevertheless, the use of stringent inclusion criteria, random allocation, and standardized clinical procedures contributed to maintaining strong internal validity and ensuring reliable outcomes. A practical challenge encountered during the trial was the difficulty in recruiting participants with four molars affected by Class II carious lesions—a requirement for implementing a split-mouth design within the study’s stringent eligibility framework^57^.

The initial sample size calculation was based on an estimated event rate (prevalence). However, identifying small to moderate variations in continuous volumetric wear across the four groups would have required a larger sample. Therefore, although the current study had sufficient power to detect large effects (as observed for the injectable material), it may not have been adequately powered to reveal smaller differences among the other materials.

## Conclusion and clinical relevance

After three years of clinical service, all tested bulk-fill resin composites—whether body, preheated, sonic-activated, or injectable—showed excellent clinical performance and comparable wear resistance. The absence of significant differences among materials suggests that modern bulk-fill composites can be safely used in posterior Class II restorations, providing satisfactory functional results when applied under proper adhesive and restorative protocols.

### Clinical recommendation

The result of the current study indicates that *modern bulk-fill resin composites* can be confidently used for posterior Class II restorations, offering predictable outcomes and simplified clinical application. Clinicians may select among these bulk-fill resin composites according to their handling preference and case requirements, as all demonstrated stable wear behavior and favorable clinical outcomes over the follow-up period. Strict adherence to manufacturer instructions and appropriate adhesive application remains essential to ensure long-term success.

## Data Availability

The datasets in the current study are available from the corresponding author (Badria Goda) on reasonable request.
